# S-equol status modulates skin response to soy isoflavones in postmenopausal women: results from a randomized placebo-controlled pilot trial

**DOI:** 10.3389/fnut.2025.1671835

**Published:** 2025-11-03

**Authors:** Vineetha Vijayakumar, Eric Climent, María Enrique, Araceli Lamelas, Beatriz Álvarez, Empar Chenoll, Malwina Naghibi, Richard Day

**Affiliations:** ^1^ADM R&D Medical, ADM Health & Wellness, London, United Kingdom; ^2^ADM R&D Center-Valencia, ADM Health & Wellness, Parc Científic Universitat de València, Valencia, Spain

**Keywords:** postmenopausal women, skin health, soy isoflavone, undereye wrinkle, skin barrier function, skin hydration, age

## Abstract

**Background/objectives:**

Soy isoflavones may benefit skin health in postmenopausal women, potentially via S-equol, a gut-derived metabolite with enhanced estrogenic and antioxidant activity.

**Methods:**

Sixty-six postmenopausal women received either Novasoy®400 (*n =* 33), a soy isoflavone extract, or identical placebo (*n =* 33) for 12 weeks. Skin parameters, including crow’s feet and under-eye wrinkles, hydration, barrier function, and skin colour were assessed at baseline, D42 and D84. Urinary isoflavone metabolites, including S-equol, were measured at each time point.

**Results:**

Crow’s feet wrinkle roughness decreased by 5.6% in the Novasoy®400 group versus a 1.6% increase in the placebo group, this difference was not statistically significant. However, urinary biomarker analysis identified 46.9% of women in the Novasoy®400 group and 15.6% in the placebo group as S-equol producers. In exploratory regression analysis limited to S-equol producers, higher urinary S-equol levels were significantly associated with improved under-eye wrinkle parameters and transepidermal water loss (TEWL) at D42. Multivariable model adjusting for age, soy intake, phototype and their interactions confirmed that older women experienced greater improvements in undereye wrinkles and barrier function in response to increased S-equol levels. Skin hydration showed a modest but statistically significant association with S-equol when combining data from both D42&84, indicating a potential cumulative effect.

**Conclusion:**

Soy isoflavone supplementation increased urinary S-equol concentrations and was associated with improvements in under-eye wrinkle appearance, skin hydration, and barrier function—particularly among older postmenopausal women identified as S-equol producers. Findings suggest region-specific, time-sensitive effects dependent on S-equol levels.

**Clinical trial registration:**

https://clinicaltrials.gov/study/NCT06047145, NCT06047145.

## 1 Introduction

Menopause triggers a decline in oestrogen levels, causing various physiological alterations, including accelerated skin aging (1). The positive effects of oestrogen replacement have been demonstrated by studies showing that hormone replacement therapy (HRT) improves the skin of postmenopausal women (2, 3). However, HRT is not always recommended due to contraindications and concerns about potential side effects (4). As such, plant-derived compounds that share structural similarities with oestrogen and can mimic oestrogenic activity, such as phytoestrogens, have garnered interest for their potential role in physiological processes linked to oestrogen depletion.

Isoflavones are one of the major classes of bioactive phytochemicals present in the soybean (5) and are the most common phytoestrogen in the human environment (6). Multiple studies have demonstrated that taking soy isoflavone supplements during menopause can improve vasomotor symptoms, reduce hypertension, attenuate bone mineral density (BMD) loss and improve cognitive function (7–9). In addition, both topical and oral soy isoflavone supplementation have shown some beneficial effects on cutaneous ageing parameters, such as wrinkle depth, hydration and skin barrier function (10). However, few studies have examined the effects of orally administered soy isoflavones on skin health in postmenopausal women, specifically.

**Figure 1:**
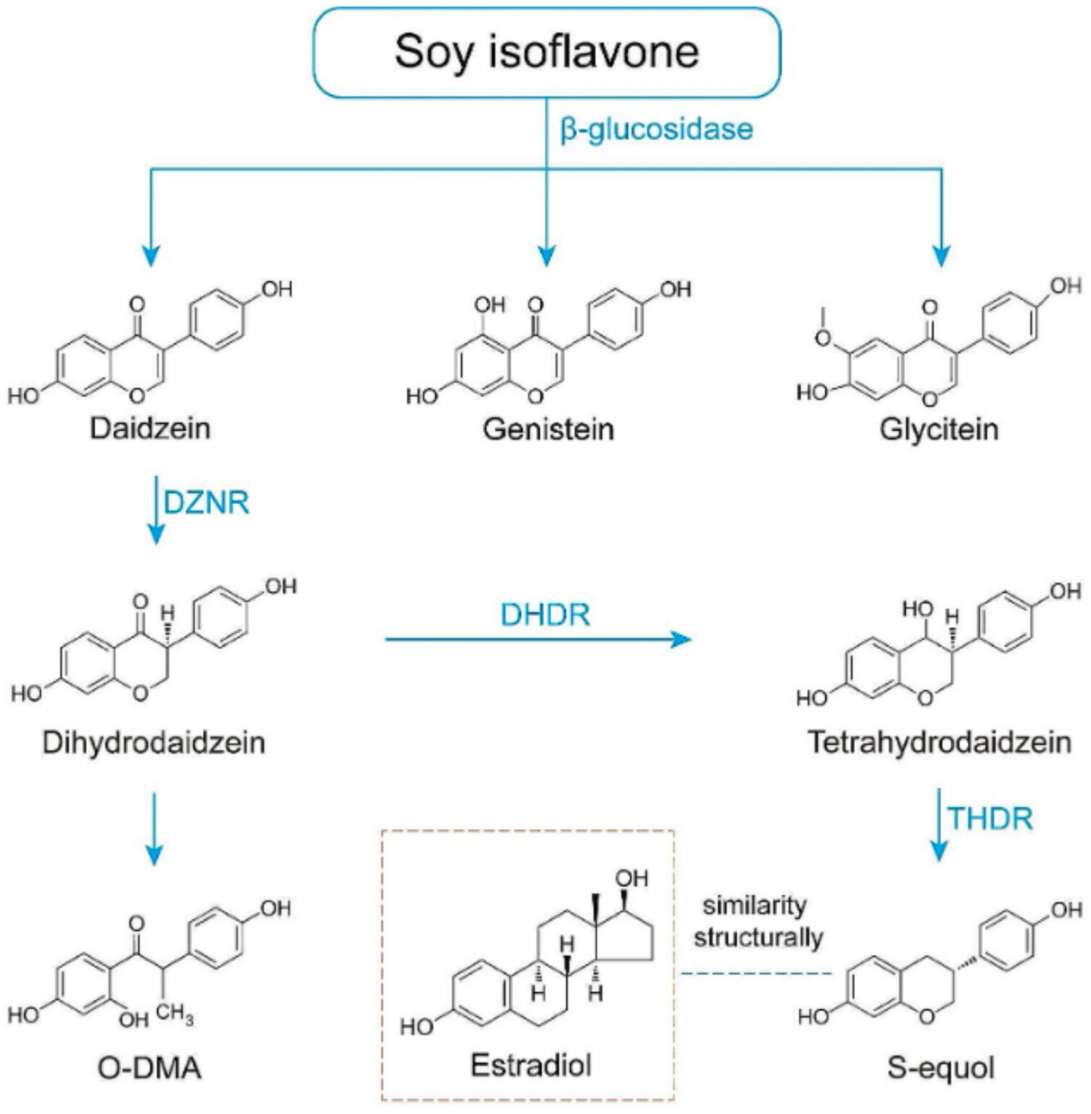
Schematic representation of equol biosynthesis and the similarity of equol to estrogen. DZNR: Daidzein Reductase; DHDR: Dihydrodaidzein Reductase; THDR: Tetrahydrodaidzein Reductase.

The biological activity of isoflavones is largely dependent on their constituent aglycones, genistein and daidzein, and their metabolites (Figure 1) (11). Of these metabolites, S-equol, the final product of daidzein biotransformation, is considered the most biologically potent due to its greater affinity for certain oestrogen receptors (12, 13). The conversion of daidzein to S-equol is a complex gut microbiota-dependent process (14). This transformation is mediated by specific bacterial species in the gut, including *Eggerthellaceae*, and *Bifdobacteriaceae* family, such as *Slackia isoflavoniconvertens* (15), *Adlercreutzia equolifaciens*, *Eggerthella* sp. YY7918 (16), and *Lactococcus garvieae* 20–92. These bacteria possess the enzymatic machinery necessary to transform daidzein into dihydrodaidzein and subsequently into S-equol. In the case of *L. garvieae* 20–92, this capability is likely due to a horizontal gene transfer (HGT) event where the genes were acquired from an *Eggerthellaceae* species (15). However, not everyone possesses the necessary gut bacteria to produce S-equol. Some estimates suggest that in East Asian populations 50–80% of individuals are S-equol producers, whereas this rate is only 20–35% in Western populations - a discrepancy largely attributed to the lower dietary exposure to soy (14, 17). It has been proposed that the ability to produce S-equol is key for achieving clinical benefits from soy isoflavones (18). Indeed, some studies have shown that postmenopausal women who are S-equol producers experience greater improvements of menopausal symptoms and bone health (19, 20). Therefore, S-equol should be considered when evaluating the impact of isoflavone supplements on skin health as well.

The aim of this study is to expand upon existing evidence of the benefits of soy isoflavones in postmenopausal women with Fitzpatrick skin types I–IV, by examining the effects of a 12-week supplementation period on parameters of skin health and determining whether these effects are S-equol dependent. We hypothesize that soy isoflavone supplementation will improve skin health parameters, and that these effects will be more pronounced in individuals who produce S-equol.

## 2 Materials and methods

### 2.1 Study design, recruitment and randomization

**Figure 2:**
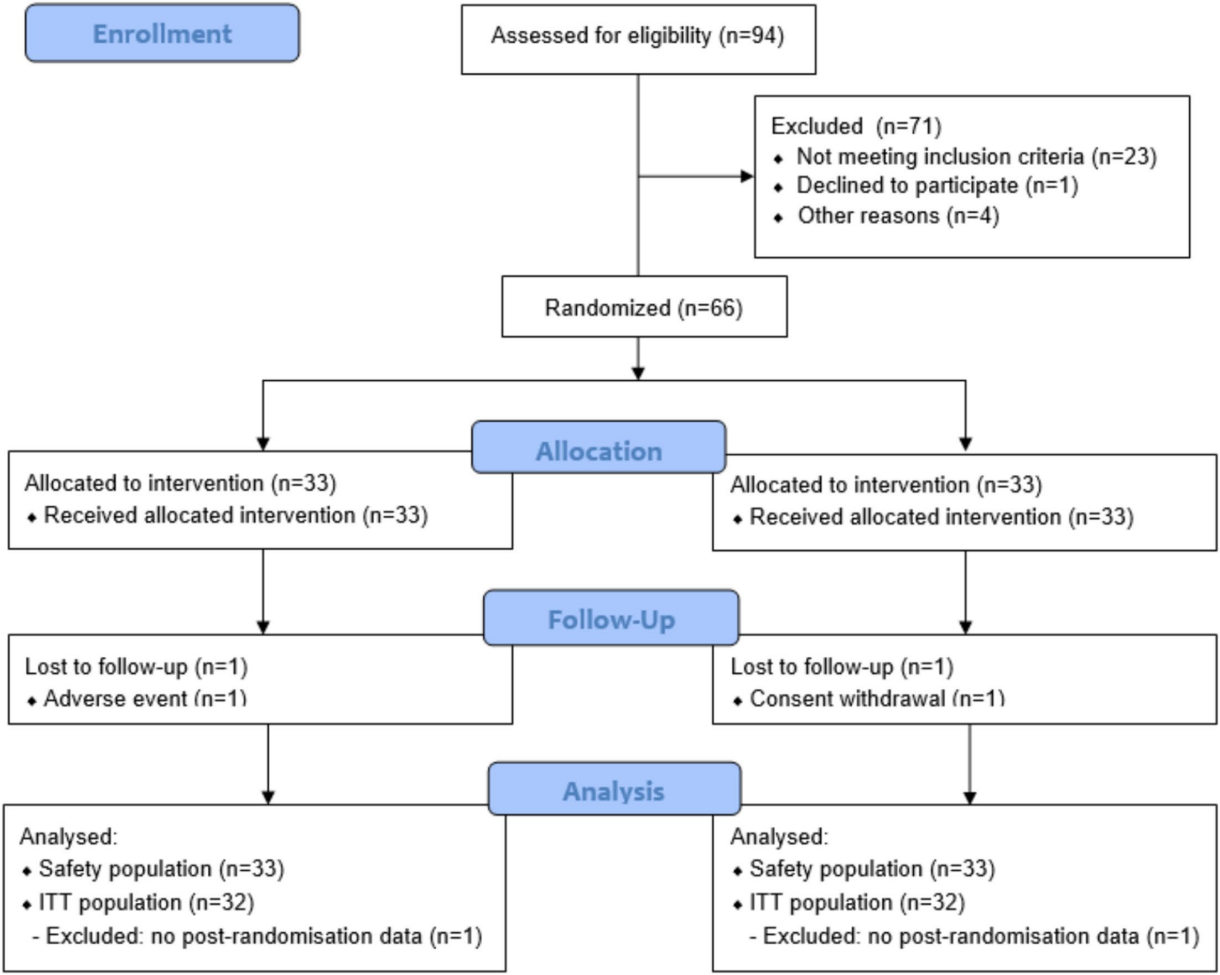
CONSORT flow diagram.

This triple-blind, randomized parallel-arm placebo-controlled pilot trial was conducted in Villeurbanne, France at Eurofins Laboratoire Dermscan. The study protocol and participant consent were reviewed and approved by the ethics committee from the Comité de protection des personnes Est I, Dijon, France (2023-A01033-42). The study was conducted in accordance with the Declaration of Helsinki and Good Clinical Practice (GCP) and registered at www.clinicaltrials.gov (NCT06047145). Written informed consent was obtained from all participants. Participants were randomly allocated to intervention arms on a 1:1 ratio, stratified by smoking status, using random permuted blocks of *n =* 2 generated using SAS v9.4. Given the small number of smokers enrolled, no subgroup analysis by smoking was performed. The intervention was in the form of capsules containing 200 mg Novasoy®400 (ADM, Illinois, USA), providing 80 mg soy isoflavones in glycoside form. Placebo capsules contained microcrystalline cellulose and were matched in taste, size and appearance. All capsules were manufactured in ADM’s facility in Somerset, UK. Participants were instructed to take one capsule daily in the morning for 12 weeks (84 days). Compliance was monitored through daily logs and capsule counts at each visit. Study visits occurred at screening, baseline (Week 0 or D0), mid-point (Week 6 or D42) and end-of-intervention (Week 12 or D84). Skin assessments were performed at each visit on the right-hand side of the face by trained personnel using calibrated equipment. Participants were asked not to apply any product to the test areas from the previous evening. On D0 first morning mid-stream urine samples were collected and then subsequently at each visit and stored at −20 ^⸰^C until analysis. A validated soy food frequency questionnaire (FFQ) was administered at Week 0 as an estimate of habitual soy food consumption (21). In total, 94 individuals were assessed for eligibility and 66 participants were randomized to either Novasoy®400 or placebo. A CONSORT diagram is presented in Figure 2. Any adverse events of the intervention were noted throughout the study.

### 2.2 Inclusion and exclusion criteria

Sixty-six healthy postmenopausal women (defined as last menstrual period >12 months ago), aged 40–65 years, were included if they were Fitzpatrick phototypes I-IV and had crow’s feet wrinkle grades 2–5 (Bazin’s scale). Exclusion criteria included (i) family history of breast, uterine or ovarian cancer, (ii) personal history of cancer, (iii) any skin or systemic disease, (iv) soy allergy, (v) current use of an anti-coagulant, hormone or osteoporosis treatment (except vitamin D or calcium), (vi) current use of supplements used to treat menopause symptoms, (vii) invasive cosmetic treatments on the test areas in the preceding 12 months, (viii) topical corticosteroids in the past 2 weeks or retinoids or immunosuppressors in the past 3 months on the test areas, (ix) intensive exposure to sunlight/UV within the past month and planned during the study without adequate protection, (x) excessive alcohol (>2 glasses of wine/day) or tobacco consumption (>10 cigarettes/day). The study was performed between October 2023 and February 2024, a period of low UV exposure in northern Europe. Participants were excluded if they reported intensive UV exposure within the previous month or planned such exposure during the study without adequate protection. They were instructed to maintain their usual skincare routines and to avoid applying any topical products to the test areas from the evening before each visit. UV exposure and skincare use between visits were not formally monitored.

### 2.3 Facial imaging and skin biophysical measurements

The primary outcome was change in crow’s feet wrinkles parameter Ra (average roughness) between D0 and D84, measured using fringe projection system Phaseshift Rapid In-vivo Measurement Of Skin optical 3D (PRIMOS®3D). Secondary outcomes included changes in crow’s feet wrinkles parameter Rz (average height of the roughness depth/average relief) and Rt (maximum height of the roughness/maximum relief amplitude), undereye wrinkles parameters Ra, Rt and Rz (projection system PRIMOS 3D), skin hydration (measured using Corneometer® CM825 on the forearm), skin color (parameters a* green-red spectrum), b* (blue-yellow spectrum), C* (saturation) and L* (clarity), measured using MINOLTA CM700-d Spectrophotometer® on the cheekbone and skin barrier function (trans-epidermal water loss (TEWL), Aquaflux®AF200 on the forearm) between D0, D42 and D84.

### 2.4 Urinary isoflavone metabolites levels

On measurement days, the first urine of the day was collected in sterile polypropylene ClearLine® containers and stored at −20 °C until analysis. Urinary levels of soy isoflavones (daidzein, genistein, glycitein, dihydrodaidzein, O-desmethylangolensin, and equol) were evaluated by liquid chromatographic separation performed on an Acquity Arc system coupled to an Acquity QDa single quadrupole mass detector (Waters Corporation Milford, MA, USA), controlled by Empower3 (v. 7.4). Urine samples underwent hydrolysis following Saha and Kroon’ protocol (22), with modifications. Briefly, 200 μL of sodium phosphate buffer (pH 6.8), 80 μL of the hydrolytic enzymes *β*-glucuronidase/sulfatase (β-glucuronidase from *Helix pomatia* in phosphate buffer, at 10,000 and 550 U/mL of β-glucuronidase and sulfatase activity, respectively) and 10 μL of taxifolin (10 μg/mL in DMSO) were added to 200 μL of urine or water (negative control). After 1 min shaking at 1200 rpm, samples were incubated for 2 h at 37 °C without shaking. Then 570 μL N, N-dimethylformamide and 40 μL formic acid were added and samples shaken during 30 s each 5 min for 10 min. Finally, samples were centrifuged at 13,000 rpm for 15 min at 3 °C and stored at −20 °C. For the determination of isoflavones the methodology of Palma-Duran et al. (23) was followed, with modifications. Isoflavone separation was performed on a Luna C18 (250 × 4.6 mm, 5 μm) column with a matching guard cartridge (Phenomenex, CA, USA). Mobile phases A and B consisted of 0.1% formic acid in milli-Q filtered water and acetonitrile, respectively. The separation was carried out under gradient conditions at 0.5 mL/min as follows: 0–5 min at 5% B, 5–8 min up to 35% B, 8–28 min up to 60% B and 28–34 min up to 95% B, with a final cleaning step of 6 min at 95% B and re-equilibration to the initial conditions over 10 min before the next analysis. The column was maintained at 35 °C, samples at 15 °C. Peaks were detected with a single quadrupole mass detector equipped with an electrospray ionization (ESI) interface (mass range 2-1250 m/z). Source and probe temperatures were set to 120 °C and 600 °C, respectively. Capillary voltage was 0.8 kV, and cone voltage 15 V. The sampling rate was 8 points/s, with a gain of 10. Compounds were monitored in Single Ion Recording (SIR) positive mode between 8-35 min (molecular ions were detected at m/z 255.2, m/z 271.2, m/z 243.2 and m/z 257.2 for daidzein, genistein, S-equol and dihydrodaidzein, respectively). Standard curves were used for quantification. The low limit of quantification for S-equol and genistein were 0.35 ppm and 0.04 ppm, respectively.

### 2.5 Statistical analysis

As this was a pilot study to assess effect size in the studied parameters, no formal sample size calculation was conducted. Based on recommendations for estimating the standard deviation of wrinkle depth within approximately 20% precision to support future sample size calculations, 30–35 participants per group was considered adequate (24). We aimed to enroll 66 participants to have at least 30 completers per arm, allowing for 10% drop-out. Primary and secondary endpoints were evaluated for normality and analysed using mixed linear models including factors for group, time and group-time interaction and a random intercept by participant. These analyses were performed on the intent-to-treat (ITT) dataset, comprised of all participants who had any post-randomization data, in accordance with ICH-GCP E9 (25). Safety analyses were performed on the entire dataset. Prevalence of metabolites after intervention was tested with Cochran Test. As exploratory analyses, skin parameters were also compared between S-equol producers and non-producers using Student’s *t*-test when data was confirmed to be normally distributed with a Shapiro test, and with Mann–Whitney U Test if not. In addition, multiple linear regression models were used to examine the relationship between each skin parameter and S-equol concentration more precisely while controlling for relevant covariates and interaction terms. All numeric covariates were centered prior to inclusion in the model. These analyses were performed in R v4.3.3, using the *step* function for backward stepwise variable selection to remove unnecessary predictors, using AIC criteria (26). *p*-values of the linear regressions were corrected following Benjamini-Hochberg procedure to reduce the risk of type I errors. The final model retained the following covariates: age (centered), dietary soy intake (centered), product consumption and phototype, along with their respective interaction terms.

$$\begin{array}{l} \mathrm{Yi}\sim \log(S-\text{equol})+\mathrm{Age}+\text{Dietary}\;\mathrm{soy}+\text{Phototype}+\\ \text{Supplementation}+\log(S-\text{equol})|\mathrm{Age}+\log(S-\text{equol})\\ |\text{Dietary}\;\mathrm{soy}+\text{Supplementation}|\mathrm{Age}+\text{Dietary}\;\mathrm{soy}|\mathrm{Age} \end{array}$$

Most of the regression results met the assumptions required for valid statistical inference, that were obtained with lmtest v0.9 library, including normality (Calculated with Shapiro test), zero mean, constant variance (Calculated with Breusch-Pagan test), and uncorrelated residuals (Calculated with Durbin-Watson test). Exceptions included: Skin hydration (Day 84), transepidermal water loss (post-intervention) and clarity (post-intervention), which violated the normality assumption, Undereye wrinkles (Day 84), transepidermal water loss (Day 42), which did not meet the assumption of uncorrelated residuals. For those skin parameters, heteroskedasticity-consistent standard errors were calculated via the vcovHC method of the sandwich v3.1 library. This adjustment improves the reliability of the coefficient-level inference under conditions of non-constant variance or correlated residuals. Significance alpha level was set at 0.05 for all analyses.

## 3 Results

### 3.1 Participant characteristics

Sixty-six women were randomised, of which 64 (*n =* 32 Novasoy®400, *n =* 32 placebo) had at least one post-randomisation visit and were included in the ITT population (Figure 2). The groups were comparable in terms of baseline characteristics, with duration of menopause numerically higher in the Novasoy®400 arm but not statistically different (Table 1). The median consumption of soy foods in both arms was low (0.46 weekly servings) which is in line with previous reports of soy consumption in the French population (27). However, the mean range varied considerably between groups with the placebo arm showing higher soy consumption through diet than Novasoy®400 group, an effect which was driven by one outlier in the placebo group.

**Table 1:** Baseline demographic characteristics.

		Novasoy® 400 (*n =* 32)	Placebo (*n =* 32)	*p*-value
Age	Years, M (SD)	58.85 (4.54)	56.82 (4.19)	0.064
Menopause	Months, M (SD)	99.79 (56.44)	79.06 (51.30)	0.123
Phototype	II, *n* (%)	14 (42.4%)	14 (42.4%)	1.000
	III, *n* (%)	19 (57.6%)	19 (57.6%)	
Ethnicity	Asian, *n* (%)	0 (0.0%)	1 (3.0%)	0.368
	White, *n* (%)	32 (97.0%)	32 (97.0%)	
	Mixed, *n* (%)	1 (3.0%)	0 (0.0%)	
Weight	Kg, M (SD)	64.76 (13.37)	60.15 (11.00)	0.131
BMI	Kg/m^2^, M (SD)	24.00 (4.73)	22.42 (3.90)	0.144
Smoker	No, *n* (%)	27 (81.8%)	28 (84.8%)	0.741
	Yes, *n* (%)	6 (18.2%)	5 (15.2%)	
Soy food,	Mdn. (min-max)	0.46 (0.00–8.00)	0.46 (0.00–53.00)	0.917
Weekly servings	Mean (SD)	1.68 (2.28)	3.30 (9.36)	
Crow’s feet (Bazin scale)	Grade 2, *n* (%)	17 (51.5%)	19 (57.6%)	0.724
	Grade 3, *n* (%)	13 (39.4%)	10 (30.3%)	
	Grade 4, *n* (%)	3 (9.1%)	4 (12.1%)	

### 3.2 Tolerability and compliance

No serious adverse events occurred, with 43 adverse events reported in the placebo group and 29 in the Novasoy®400 group. Only two participants experienced adverse reactions, including one in the placebo arm (bloating) and one in the Novasoy®400 arm (spasmodic stomach ache, diarrhoea), who was withdrawn from the study due to symptom persistence. Product compliance was excellent with 100 and 101% in the Novasoy®400 and placebo groups, respectively.

### 3.3 Skin health parameters

**Figure 3:**
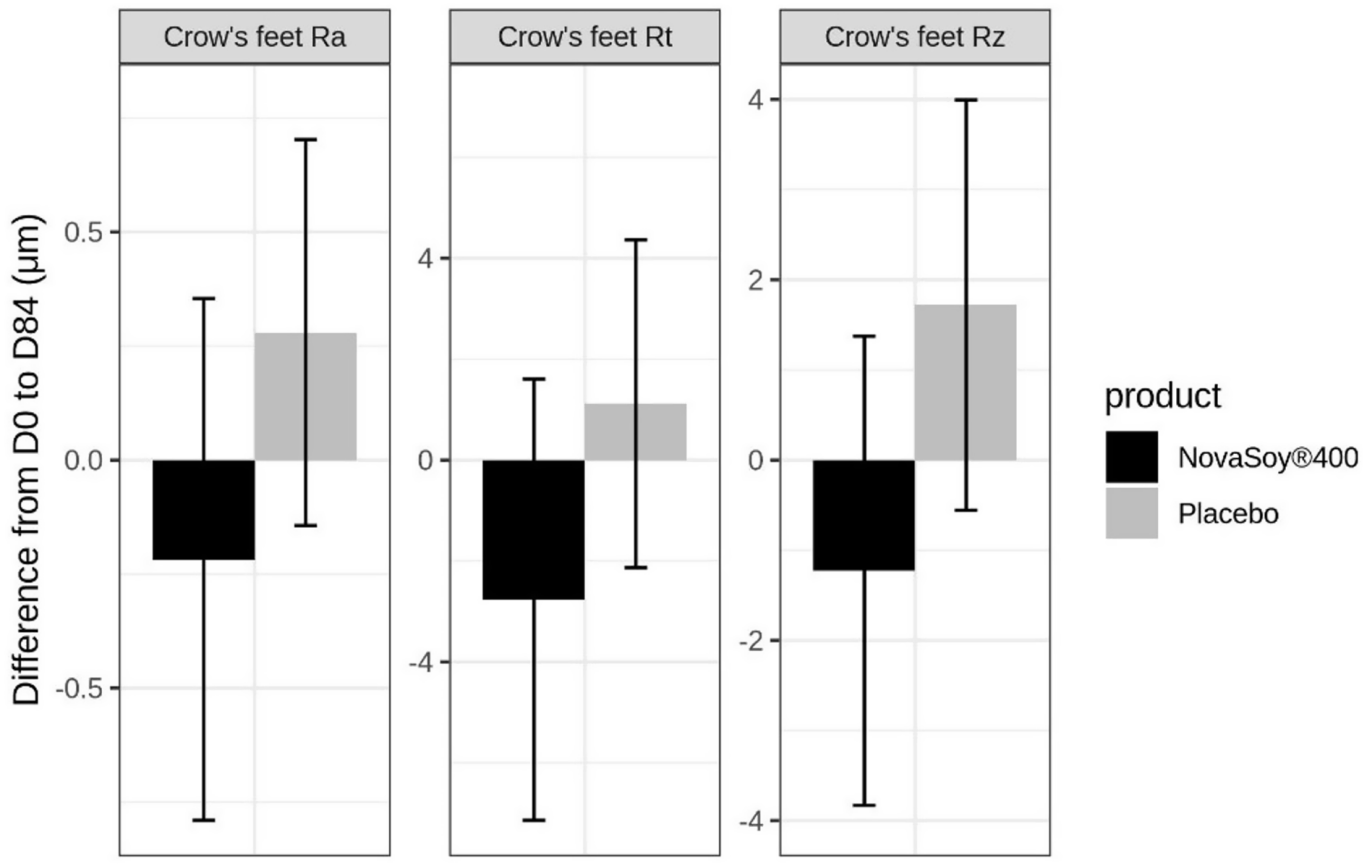
Change from baseline to end of trial in crow’s feet Ra, Rt and Rz parameters. Ra = Average roughness, Rz = Average height of the roughness, Rt = Maximum height of the roughness. Error bars are standard error of the mean.

Overall, the Novasoy®400 group showed a 5.62% reduction for the average crow’s feet roughness (Ra) suggesting an improvement in wrinkle roughness whereas the placebo group had a 1.65% increase in average wrinkle roughness (Figure 3 and Supplementary Table 1). Although, this difference did not reach statistical significance (*p =* 0.352), even after correcting for duration of menopause (*p =* 0.354). At Day 84, the direction of improvement in crow’s feet average height (Rz) and maximum height (Rt) in the Novasoy®400 group followed a similar pattern to that observed for average roughness (Ra)—all showing a reduction, indicating a consistent improvement in wrinkle depth and surface texture. However, there were no significant between-group differences for any of the other skin health parameters; undereye wrinkles (Ra, Rz, Rt), skin hydration, TEWL or skin colour parameters (a, b, C*, L*) at either D42 or D84 (Supplementary Table 1).

### 3.4 Urinary isoflavone metabolites

**Figure 4:**
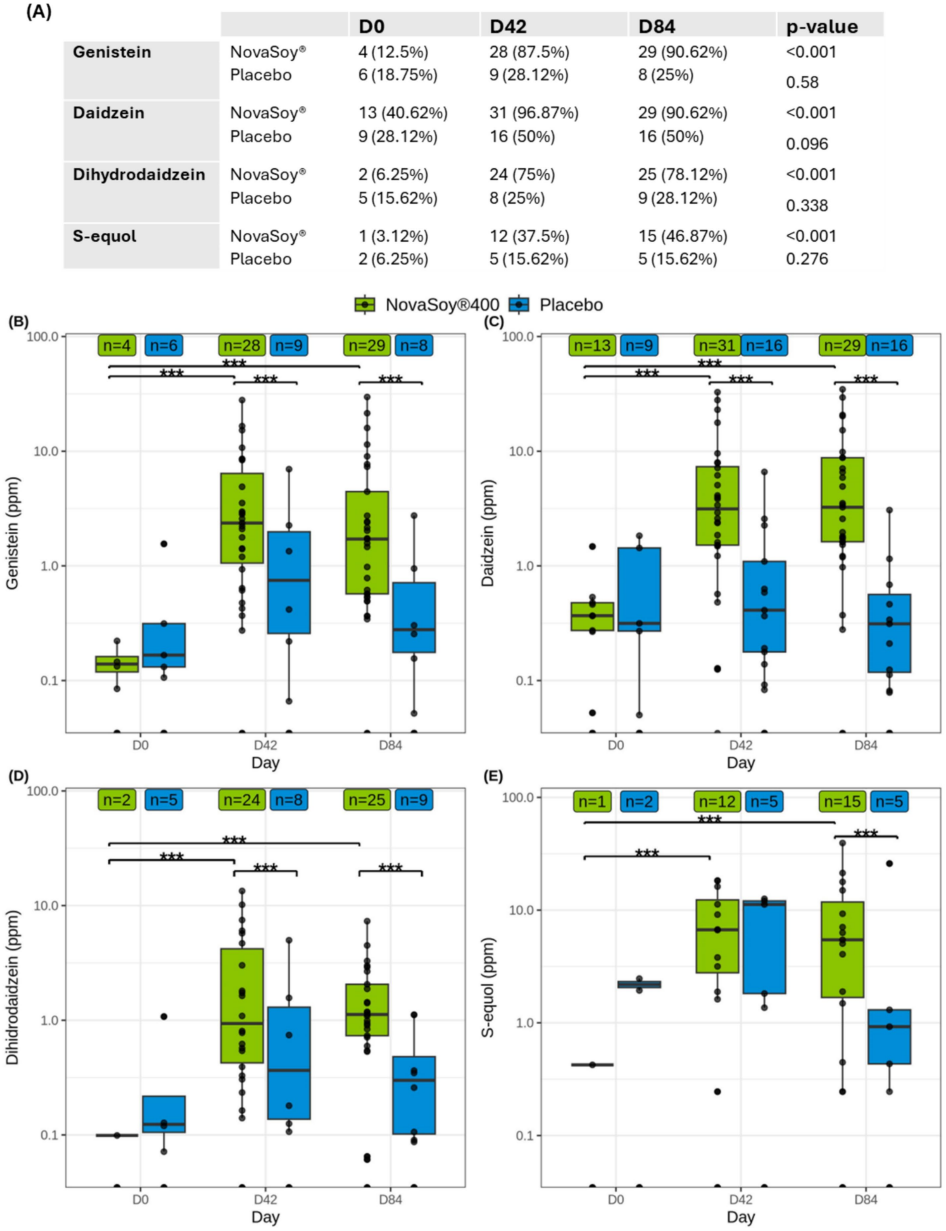
**(A)** Number and percentage of participants with detectable levels of genistein, daidzein, dihydrodaidzein, and S-equol in urine at different visits (D0: day 0, D42: day 42, D84: day 84) by group. Statistical significance in the proportions across different time points was assessed using the Cochran test. Concentrations of **(B)** genistein, **(C)** daidzein, **(D)** dihydrodaidzein and **(D)** S-equol in urine at different visits (D0: day 0, D42: day 42, D84: day 84) by group. Statistical significance between different time points and the baseline (D0), or between groups was assessed using the Mann–Whitney-Wilcoxon test. n: Number of subjects with detectable metabolites at each time point and group. *p < 0.05, **p < 0.01, ***p < 0.001.

At baseline, both the Novasoy®400 and placebo groups showed similar prevalence and concentration of genistein, daidzein, dihydrodaidzein, and S-equol in urine samples (Figures 4A–D; Supplementary Table 2).

Following supplementation with Novasoy®400, there was a statistically significant increase in the number of women with detectable levels of all four soy metabolites over time, as assessed by Cochran’s Q test (*p <* 0.05) (Figure 4A). By Day 84, detection rates in the Novasoy®400 group reached 90.62% for genistein (*n =* 29), 90.62% for daidzein (*n =* 29), 78.12% for dihydrodaidzein (*n =* 25), and 46.87% for S-equol (*n =* 15), while the placebo group showed no significant changes.

In addition, supplementation with Novasoy®400 significantly increased the concentration of all four metabolites from D0 to D84, as determined by the Wilcoxon test (*p <* 0.05) (Figures 4B*–*E). Notably, genistein level increased by 30-fold from baseline within the Novasoy®400 group (*p <* 0.001) and was 8 times higher than the placebo group at D84 (*p <* 0.05) (Figure 4B; Supplementary Table 2). Similarly, daidzein and dihydrodaidzein concentrations were 16.6- and 4.3-fold higher, respectively, compared to the placebo group producers at D84 (*p <* 0.05) (Figures 4C,D; Supplementary Table 2). S-equol levels were also 1.56 times higher in the Novasoy®400 group than in equol producers in the placebo group at D84, driven by a within-group increase from 0.42 ppm ± 0.00 at D0 to 9.0 ± 10.64 ppm at D84. Due to the low number of producer subjects in the NovaSoy ®400 group at baseline (*n =* 1), within group statistical testing was not applicable. No significant change in S-equol was observed in the placebo group (*p =* 0.38) (Figure 4E; Supplementary Table 2).

Given that S-equol production depends on the gut microbiome and that dietary soy intake varied widely among participants, we focused on the subset of women who showed detectable S-equol levels in their urine at D84 (i.e., S-equol producers *n =* 15 in the Novasoy®400 group and *n =* 5 in the placebo group; see Figure 4). This corresponds to 50% of women in the Novasoy®400 group being S-equol producers. Among these individuals, those supplemented with Novasoy®400 produced 1.5 times more S-equol than their counterparts in the placebo group, whose isoflavone exposure derived solely from dietary sources.

### 3.5 Exploratory regression analysis of S-equol and skin parameters

An exploratory analysis was conducted to understand the relationships between S-equol and skin parameters at each time point. For this analysis, we combined participants from Novasoy®400 and placebo group who were S-equol producers and who had complete skin parameter data for each time point (n_D42_ = 14, n_D84_ = 17 and n_D42&84_ = 31).

#### 3.5.1 Correlation analysis

**Figure 5:**
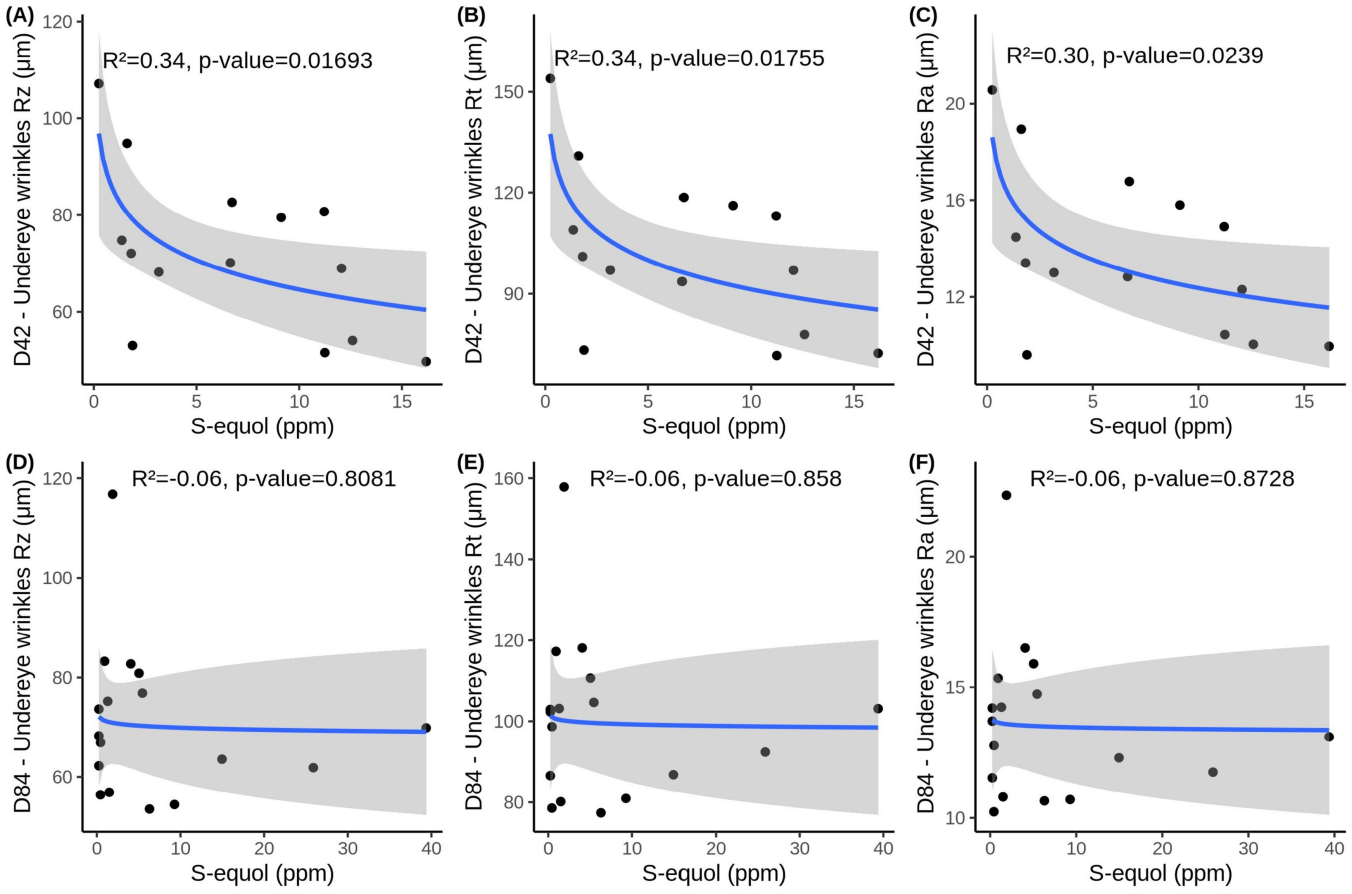
Simple regression analysis between urinary S-equol levels and Undereye wrinkle parameters **(A)** Rz, **(B)**, Rt **(C)**, Ra at D42 and **(D)** Rz, **(E)** Rt, **(F)** Ra at D84. Data includes women that were used in the multiple regression model and had any detectable amount of S-equol in that time point (n_D42_ = 14, n_D84_ = 17). Adjusted R^2^ and *p*-values obtained with F-test are depicted in the figures.

On Day 42, Figures 5A–C show a clear inverse, non-linear relationship between S-equol levels and undereye wrinkle severity, with higher S-equol concentrations associated with lower wrinkle scores (Rz, Rt, and Ra). These associations are supported by moderate R^2^ values, with approximately 30–34% of the variability in wrinkle depth, total height, and surface roughness explained by S-equol levels. These statistically significant results suggest, a moderate and meaningful relationship and indicate a potential beneficial effect of S-equol on undereye wrinkle appearance at this earlier time point. Of note, the most substantial improvements in wrinkle appearance occurred within the 0–5 ppm range, after which the effect plateaued, indicating that a maximum level of improvement may have been reached, after which further increases in S-equol provided diminishing additional benefit. By Day 84, this relationship was no longer observed (Figures 5D*–*F), nor was it apparent when both D42&D84 time points were combined (Supplementary Table 3), potentially reflecting a saturation point in the skin’s responsiveness to S-equol or the achievement of a new equilibrium in wrinkle morphology.

**Figure 6:**
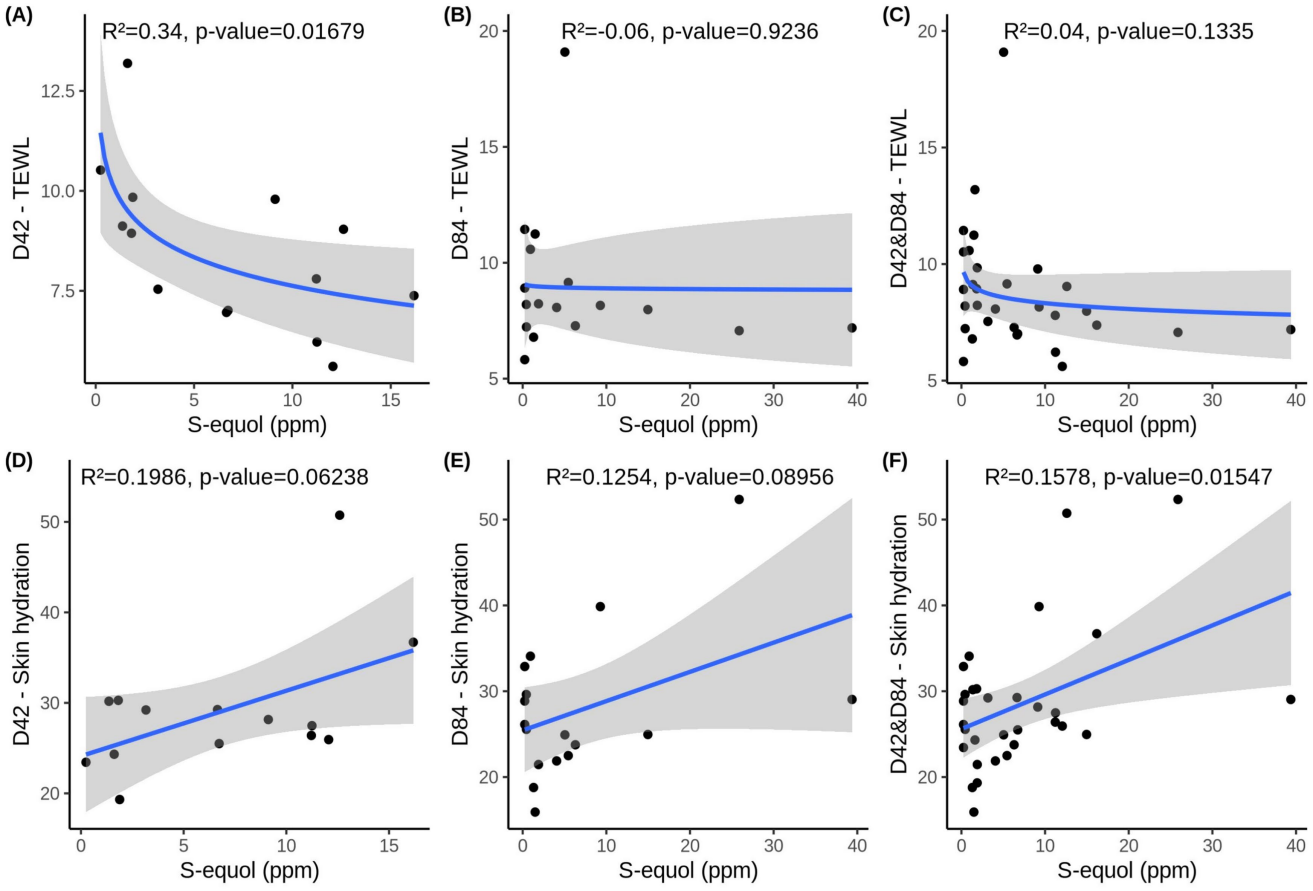
S-equol levels and two skin health parameters—TEWL (transepidermal water loss) **(A)** at Days 42, **(B)** at Days 84 and **(C)** over time (D42&D84) and skin hydration D) at Days 42, **(E)** at Days 84 and **(F)** over time (D42&D84). Data includes women that were used in the multiple regression model and had any detectable amount of S-equol in that time point (n_D42_ = 14, n_D84_ = 17, n_D42&D84_ = 31). Adjusted R^2^ and *p*-values obtained with F-test are depicted in the figures.

When examining TEWL at Day 42, the simple regression model reveals a relationship similar to that observed for undereye wrinkles, with a logarithmic association between S-equol levels and TEWL (Figure 6A). The model explained approximately 34% of the variability and reached statistical significance, indicating that higher S-equol levels are associated with lower TEWL, with similar substantial improvements occuring within the 0–5 ppm range of S-equol. However, this association was not maintained at D84 or combined D42&84 (Figures 6B,C). This indicates that the association may be time-dependent and non-linear, and that the effect of S-equol on TEWL is more evident at D42 than when data from both time points are combined.

In contrast, skin hydration showed a linear relationship with S-equol. At D42 and D84, there is a modest association, with S-equol explaining 13% of the variability in hydration levels. However, this relationship did not reach significance (D42: *p =* 0.06238, D84: *p =* 0.0896) (Figures 6D,E). In contrast, when combining D42&84 (Figure 6F), the association becomes statistically significant (*p =* 0.0155), and the model explains approximately 16% of the variability in skin hydration. These findings suggest that S-equol may have a modest positive effect on skin hydration and that pooling data across time points increases the power to detect this relationship.

For the remaining skin parameters, including crow’s feet wrinkles and skin colour parameters, there was no clear linear or logarithmic relation found (the R^2^ and *p*-values are presented in Supplementary Table 3).

Overall, while simple regression model using S-equol as the sole predictor reveals some statistically significant associations with undereye wrinkle parameters, TEWL, and skin hydration, the proportion of variability explained remains modest across all outcomes. This suggests that S-equol alone is not sufficient to fully account for the observed differences in skin health metrics. To better understand and predict these outcomes, it is likely that additional variables—such as age, dietary soy intake, and skin phototype need to be incorporated into a more comprehensive multivariable model.

#### 3.5.2 Multivariable model adjusted for age, soy intake, and phototype

Multiple linear regression analysis examined the relationship between the skin parameters and various predictor variables (age, dietary soy intake, and skin phototype) and their interactions at D42, D84, and for both timepoints combined D42&84. In other words, independent predictor variables or the combinations of predictor variables (interactions) were tested for their association with the respective skin parameter. Of note, we used the log-transformed S-equol values to account for the wide variation in S-equol levels, which helped enhance the accuracy and reliability of the multiple linear regression model (Supplementary Table 4). The regression models were tested for statistical assumptions (Supplementary Table 5), and heteroskedasticity-consistent standard errors were calculated via the vcovHC method in R in case they did not met them, to improve the reliability of the coefficients obtained with the inference. The regression model was statistically significant for five different skin parameters at D42, six at D84 and 11 for combined D42&84. Tables 2–4 present only the statistically significant results of this analysis and how the various predictors or covariates: log(S-equol), age, weekly soy consumption, and their interactions influence the different skin parameters.

- Day 42:

**Table 2:** Results of the multiple linear regression model showing the influence of S-equol, age and soy consumption on skin parameters at D42.

Skin parameter	Predictor/interaction	*β*	S. E.	*p-*value	Direction
Average roughness, undereye wrinkles (Ra) (Adj. R^2^ = 0.84/Model adj. *p =* 0.028)	Intercept	14.58	0.68	0	NA
	log(S-equol)	−1.34	0.39	0.019	−
	log(S-equol) × Age	−0.36	0.14	0.048	−
	Age × Weekly soy	−5.36	1.57	0.019	−
Maximum Roughness (Rt) (Adj. R^2^ = 0.89/Model adj. p- value = 0.019)	Intercept	108.09	4.1	0	NA
	log(S-equol)	−10.76	2.36	0.006	−
	log(S-equol) × Age	−2.52	0.82	0.028	−
	Age × Weekly soy	−35.61	9.43	0.013	−
	log(S-equol) × Age × Weekly soy	17.86	6.7	0.045	+
Average Height of Roughness (Rz) (Adj. R^2^ = 0.93/Model adj. *p =* 0.012)	Intercept	76.57	2.25	0	NA
	log(S-equol)	−7.63	1.3	0.002	−
	Age	1.71	0.6	0.035	+
	log(S-equol) × Age	−1.81	0.45	0.01	−
	Age × Weekly soy	−23.84	5.18	0.006	−
	log(S-equol) × Age × Weekly soy	10.75	3.68	0.033	+
Transepidermal Water Loss (TEWL) (Adj. R^2^ = 0.87/Model adj. *p =* 0.02)	Intercept	10.29	0.29	0	NA
	log(S-equol)	−1.22	0.12	0	−
	Age	−0.39	0.09	0.009	−
	log(S-equol) × Age	0.23	0.06	0.014	+
	Age × Weekly soy	−2.46	0.53	0.006	−
	log(S-equol) × Age × Weekly soy	2.59	0.38	0.001	+
Skin Colour (b*) (Adj. R^2^ = 0.62/Model adj. *p =* 0.028)	Intercept	17.32	0.35	0	NA
	Age × Weekly soy	2.7	0.58	0.001	+

**Table 3:** Results of the multiple linear regression model showing the influence of age, weekly soy, phototype III, and placebo on skin parameters at D84.

Skin parameter	Predictor/interaction	*β*	S. E.	*p*-value	Direction
Average roughness, undereye wrinkles (Ra) (Adj. R_2_ = 0.76, Model adj. *p <* 0.001)	Intercept	11.89	0.45	0	NA
	Age	−0.45	0.1	0.001	−
	Weekly soy	2.46	0.31	0	+
	Phototype III	2.48	0.77	0.001	+
	Age × Placebo	1.78	0.48	0.003	+
	Age × Weekly soy	−4.99	0.72	0	−
Maximum height of the roughness, undereye wrinkles (Rt) (Adj. R_2_ = 0.73, Model adj. *p =* 0.008)	Intercept	89.48	3.81	0	NA
	Age	−3.15	0.63	0	−
	Weekly soy	14.88	2.31	0	+
	Phototype III	14.78	5.29	0.017	+
	Age × Placebo	13.24	3.53	0.003	+
	Age × Weekly soy	−31.53	4.88	0	−
Average height of roughness, undereye wrinkles (Rz) (Adj. R_2_ = 0.71, Model adj. *p =* 0.008)	Intercept	62.70	2.90	0	NA
	Age	−2.26	0.55	0.002	−
	Weekly soy	12.75	1.86	0	+
	Phototype III	11.45	4.11	0.018	+
	Age × Placebo	9.07	3.01	0.012	+
	Age × Weekly soy	−25.11	4.15	0	−
Skin hydration (Adj. R_2_ = 0.57, Model adj. *p <* 0.02)	Intercept	23.01	1.82	0	NA
	Weekly soy	−7.83	1.74	0.001	−
	Placebo	9.61	2.47	0.002	+
Skin colour -(b*) (Adj. R_2_ = 0.5, Model adj. *p =* 0.048)	Intercept	17.89	0.38	0	NA
	log(S-equol)	−0.34	0.21	0.14	−
	Age	0.27	0.09	0.015	+
	log(S-equol) × Age	−0.15	0.05	0.008	−
	Age × Weekly soy	2.30	0.71	0.008	−
Skin colour - L* (Adj. R_2_ = 0.4, Model adj. *p =* 0.018)	Intercept	64.04	0.82	0	NA
	Phototype III	−4.11	1.12	0.004	−

Relation between the undereye wrinkles (Ra, Rt, Rz), skin hydration, skin colour - blue-yellow spectrum variables, clarity and logarithmic S-equol concentration at day 84 (D84). β = beta coefficient, S. E. = Standard error. Adj. R^2^ = coefficient of determination adjusted. − = negative direction. + = positive direction. NA = not applicable.

**Table 4:** Results of the multiple linear regression model showing the influence of S-equol, age and soy consumption on skin parameters at combined D42&D84.

Skin parameter	Predictor/interaction	*β*	S. E.	*p*-value	Direction
Average roughness, undereye wrinkles (Ra) (Adj. R^2^ = 0.27/Model adj. *p =* 0.021)	Intercept	13.86	0.57	0	NA
	Age × Weekly soy	−2.71	0.96	0.008	−
Maximum Roughness (Rt) (Adj. R^2^ = 0.20/Model adj. *p =* 0.046)	Intercept	102.40	4.16	0	NA
	log(S-*equol*) × Age	−1.19	0.58	0.047	−
	Age × Weekly soy	−14.44	7	0.049	−
Average Height of Roughness (Rz) (Adj. R^2^ = 0.25/Model adj. *p =* 0.024)	Intercept	72.40	2.92	0	NA
	Weekly soy	6.87	3.35	0.05	+
	Age × Weekly soy	−12.43	4.91	0.017	−
CrowsFeet (Ra) (Adj. R^2^ = 0.28/Model adj. *p =* 0.008)	Intercept	20.38	0.68	0	NA
	Age	0.38	0.18	0.037	+
CrowsFeet (Rt) (Adj. R^2^ = 0.30/Model adj. *p =* 0.006)	Intercept	147.52	5.14	0	NA
	Age	3.48	1.32	0.013	+
CrowsFeet (Rz) (Adj. R^2^ = 0.29/Model adj. *p =* 0.007)	Intercept	102.62	3.329	0	NA
	Age	2.14	0.85	0.018	+
Skin hydration (Adj. R^2^ = 0.52/Model adj. *p <* 0.001)	Intercept	23.21	1.36	0	NA
	log(S-*equol*)	0.57	0.15	0.001	+
	Weekly soy	−6.42	1.74	0	−
	Placebo	7.89	2.37	0.001	+
	log(S-*equol*) × Age	0.07	0.03	0.036	−
Skin colour - (L*) (Adj. R^2^ = 0.59/Model adj. *p <* 0.001)	Intercept	62.17	0.55	0	NA
	log(S-*equol*)	0.60	0.24	0.017	+
	Age	0.32	0.11	0.007	+
	Phototype III	−2.21	0.68	0.003	−
	Placebo	3.25	0.8	0	+
	log(S-*equol*) × Age	−0.17	0.08	0.034	−
	log(S-*equol*) × Age × Weekly soy	−2.22	0.76	0.007	−
Skin colour (a*) (Adj. R^2^ = 0.45/Model adj. *p =* 0.006)	Intercept	12.74	0.53	0	NA
	Age	−0.37	0.11	0.002	−
	Weekly soy	1.64	0.64	0.018	+
	Phototype III	1.55	0.65	0.026	+
	Placebo	−1.6	0.74	0.048	−
	log(S-*equol*) × Age	0.3	0.07	0.001	+
	Age × Weekly soy	−2.94	0.88	0.003	−
	log(S-*equol*) × Age × Weekly soy	2.66	0.73	0.001	+
Skin colour (b*) (Adj. R^2^ = 0.47/Model adj. *p <* 0.001)	Intercept	17.09	0.35	0	NA
	Age	0.2	0.07	0.012	+
	log(S-*equol*) × Age	−0.12	0.04	0.005	−
	Age × Weekly soy	1.87	0.49	0.001	+
Skin colour (C*) (Adj. R^2^ = 0.63/Model adj. *p <* 0.001)	Intercept	21.37	0.32	0	NA
	log(S-*equol*)	−0.34	0.14	0.02	−
	Weekly soy	1.44	0.39	0.001	+
	Phototype III	1.40	0.40	0.002	+
	log(S-*equol*) × Age	0.13	0.05	0.007	+
	log(S-*equol*) × Weekly soy	−0.60	0.27	0.033	−
	log(S-*equol*) × Age × Weekly soy	2.23	0.44	0	+

Relation between the undereye wrinkles (Ra, Rt, Rz), crow’s feet (Ra, Rt), skin hydration, skin colour (a*, b* and c*) and logarithmic S-equol concentration at day 42 and day 84. β = beta coefficient, S. E. = Standard error, Adj. R^2^ = coefficient of determination adjusted. − = negative direction. + = positive direction. NA = not applicable. Intercept: The expected value of the skin parameter when all numerical predictor variables are zero.

At day 42 the model demonstrated robust predictive power with adjusted R^2^ greater than 0.62 for five different skin parameters (Table 2) which suggests that at least one of the predictors in the model is significantly associated with the skin parameter.

Undereye wrinkles parameters (Ra, Rt, Rz) all showed a negative and statistically significant correlation with log(S-equol) (negative β regression coefficient and all *p <* 0.019) (Table 2). In other terms, higher S-equol levels were linked to fewer under eye wrinkles (all wrinkle measures—Ra, Rt, and Rz—decreased as S-equol increased), these results are in line with the simple regression model. As expected, age positively correlated with undereye wrinkles Rz (*p =* 0.035) as in, older age was linked to deeper wrinkles (Table 2).

**Figure 7:**
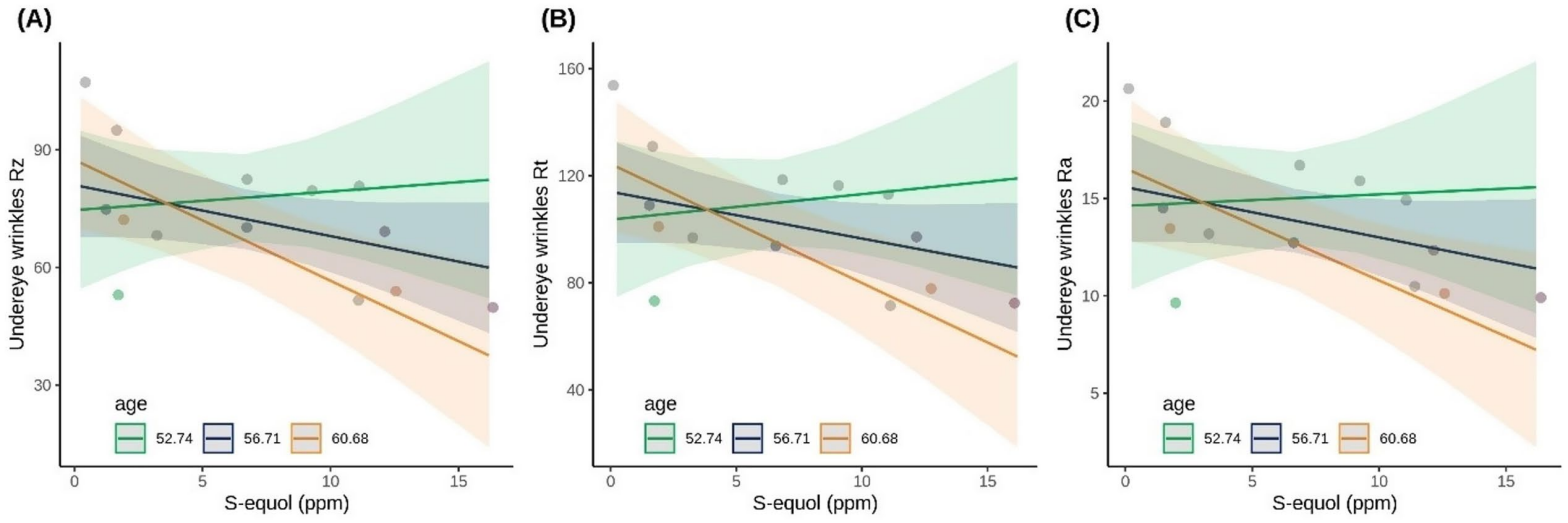
Scatterplots showing the association between S-equol concentration and undereye wrinkle parameters **(A)** Rz, **(B)** Rt, and **(C)** Ra on day 42 for S-equol producers (*n =* 14). Participants are divided into three age groups to visually interpret the interaction term of the linear model.

Additionally, significant interaction terms between log(S-equol) and age (all *p <* 0.048) and between age and weekly soy intake (all *p <* 0.019) were observed. Meaning, the effect of S-equol on wrinkle improvement might be influenced by participant’s age. Figure 7 shows that older participants experienced greater improvements in under-eye wrinkle parameters (Ra, Rz, and Rt) in response to higher S-equol levels, suggesting that age may modulate the skin’s responsiveness to S-equol. Also, the combination of age and how much soy the participant ate also influenced under eye wrinkles.

TEWL was negatively correlated with log(S-equol) (*p <* 0.001), meaning higher S-equol concentration was linked to less moisture loss so improved skin barrier. Interestingly, age was negatively associated with TEWL suggesting that older participants exhibited better skin barrier function. However, the significant interaction effect between age × weekly soy and the three way log(S-equol), age and weekly soy indicate that the relationship between age and TEWL is also influenced by soy intake and S-equol levels. If the older participants in this study were generally healthier or more responsive to soy/S-equol, this could skew the expected trend.

For skin colour b* (blue/yellow spectrum), the interaction between age and weekly soy intake variables was positively correlated (*p =* 0.001). Skin tone changes, particularly yellow hue or warm skin tones, may be influenced by age and dietary soy, though not directly by S-equol levels in this model.

- Day 84:

By contrast at Day 84, the model demonstrated less predictive power than at day 42, for the skin outcomes assessed (adjusted R^2^ between 0.4 and 0.76 for six parameters) (Table 3). At Day 84 the undereye wrinkle parameters (Ra, Rt, and Rz) no longer correlated with S-equol levels. Instead, negative correlations were observed with age (all *p <* 0.002) and the interaction between age and weekly soy intake (all *p <* 0.001). Positive associations were found with weekly soy intake (all *p <* 0.001), skin phototype III (all *p <* 0.018), and the interaction between age and placebo (all *p <* 0.012). In other terms, by day 84, the negative correlation with age and positive association between age and placebo indicates that older women who consumed Novasoy®400 appeared to have reduced undereye wrinkles compared to placebo. The analysis also indicates that both being of older age and consuming higher weekly soy were also linked to fewer wrinkles. Conversely, higher soy consumption, medium skin tone (phototype III), and older age in the placebo group were associated with increased wrinkle formation. Skin hydration was negatively correlated with the weekly soy intake (*p =* 0.001) and positively correlated with placebo consumption (*p =* 0.002) (Table 3).

Regarding skin color, older age is positively correlated with changes in the blue/yellow spectrum (b*) and having phototype III skin is negatively correlated with lightness (L*), indicating darker skin, which validates the model.

The significant interaction terms across various skin parameters highlight complex relationships where the effect of one predictor on a skin parameter can be modified by the presence of another factor, underscoring the intricate interplay of age, soy intake, phototype, and supplementation on skin characteristics. The weaker association at D84 compared to D42 suggests that the beneficial effects of Novasoy®400 supplement may have plateaued by D42, meaning the skin may have responded early and any additional changes beyond this point were minimal or variable across individuals.

- Combined D42&84:

Multiple linear regressions assessing crow’s feet parameters (Ra, Rt, Rz), red/green colour spectrum, and skin clarity were not significant at either individual time point. When combined time points were considered the model showed improved prediction accuracy for skin hydration (adjusted R^2^ = 0.52, adj.*p <* 0.001) and red/green colour spectrum (adjusted R2 = 0.45, adj.*p =* 0.006) (Table 4). On the other hand, the fit for undereye wrinkles was reduced (adjusted R^2^ between 0.2 and 0.27) compared to individual D42 and D84.

For undereye wrinkle, the combined D42&84 shows that the wrinkle parameters (Ra, Rt, and Rz) no longer correlated with S-equol levels. Instead, negative correlation was observed for the interaction between age and weekly soy intake (all *p <* 0.049). Interestingly, for maximum roughness (Rt) a significant interaction term between log(S-equol) and age (*p =* 0.047) was observed. Meaning that the effect of S-equol on average undereye roughness improvement might be influenced by participants’ age. This is in line with what was observed at D42.

For crow’s feet parameters (Ra, Rt, Rz), age was a consistent predictor of increased wrinkles across all three parameters: Ra (*p =* 0.037), Rt (*p =* 0.013), and Rz (*p =* 0.018). However, the adjusted R^2^ values for Ra (0.28), Rt (0.30), and Rz (0.29) suggest that the model explains only 28–30% of the variance in wrinkle measures highlighing that a large portion of the variation remains unexplained. No significant effects were observed for S-equol, soy intake, or their interactions in these crow’s feet models, in contrast to other areas of the face or other skin health metrics where these interventions had stronger impacts. Skin hydration was influenced by several variables when data from D42 and 84 were combined. Higher S-equol levels were positively associated with improved skin hydration (*p <* 0.001). Also, a significant interaction effect was observed between log(S-equol) and age (*p =* 0.038), implying that older individuals may experience greater hydration benefits. However, weekly soy intake was negatively associated with hydration (*p <* 0.001) and being in the placebo group was associated with a significant increase in hydration (*p =* 0.003). These data suggest that both biological age and S-equol production play key roles in improving skin hydration. However, the unexpected positive placebo effect and negative weekly soy association should be interpreted with caution given the exploratory and likely underpowered nature of these analyses, and warrant further investigation in future adequately powered studies.

In terms of skin colour parameters; L* (lightness), a* (red/green spectrum), b* (yellow/blue spectrum), and C* (chroma/saturation), all models showed modest to strong explanatory power (adjusted R^2^ = 0.45–0.63). The data shows that age, weekly soy intake, phototype III, and S-equol levels are all significant contributors to changes in skin tone. Higher urinary S-equol levels were associated with increased L*, indicating brighter or lighter skin tone, perceived as healthier or more youthful in cosmetic dermatology. Significant interactions between age, soy intake, and S-equol suggest that S-equol effect on L*, a* and C* are amplified in older women with higher weekly soy intake. Collectively, these findings highlight a nuanced, multifactorial relationship between soy metabolism, age, and skin pigmentation, positioning S-equol as a potential mediator and marker of phytoestrogen-induced changes in skin tone and radiance.

The results of the analysis of the combined timepoint confirm the complex relationships between various skin parameters and the predictors log(S-equol), age, and weekly soy consumption. The effects of S-equol, age, and weekly soy appear to vary in strength and significance at different time points, highlighting the complexity of their interactions on skin health and the need for further studies to better understand these complex interactions.

## 4 Discussion

This 12 week, randomized, triple blind placebo controlled pilot study provides novel insights into the effects of an orally administered soy isoflavone supplement on urinary metabolite profiles and skin health parameters in postmenopausal women. Supplementation with Novasoy®400 significantly increased the detection and concentration of genistein, daidzein, dihydrodaidzein, and S-equol in urine over the 84-day intervention, reflecting increased isoflavone bioavailability. In particular, 50% of participants in the Novasoy®400 group were identified as S-equol producers by Day 84, a rate notably higher than typically observed in Western populations (20–30%) (14, 17), most likely attributable to the daily standardized soy isoflavone intake during the study. It should be noted that with 64 individuals included in the ITT analysis, it is prudent to always bear in mind that this is a pilot study for this ingredient in this population, studying these endpoints. These data can be used to inform subsequent power calculations for any follow-up studies.

In terms of clinical skin outcomes, we observed a 5.6% decrease in crow’s feet wrinkle average roughness (Ra) in the Novasoy®400 group after 84 days, while the placebo group showed a 1.6% increase. Although this difference was not statistically significant, the direction and magnitude of change are consistent with prior research (19, 28). For example, a 2023 study by Rizzo et al. reported a 5.9 and 7.1% reduction in facial wrinkles compared to baseline among postmenopausal American women with Fitzpatrick skin types I–III after 16 weeks and week 24 of daily supplementation with 50 mg of soy isoflavones derived from a soy protein isolate (28). This suggests that the modest, non-significant improvement observed in our study may reflect an early-stage response, and that a longer intervention period—potentially beyond 12 weeks—may be necessary to observe significant and sustained reductions in wrinkle severity, particularly in the crow’s feet area.

Among S-equol producers, exploratory regression analyses revealed meaningful associations between S-equol levels and skin parameters, particularly at Day 42. Higher S-equol concentrations were associated with improved undereye wrinkle appearance (Ra, Rt, and Rz) and reduced transepidermal water loss (TEWL), suggesting enhancements in both dermal structure and skin barrier function. Notably, these associations appeared to be dose-responsive up to ~5 ppm of urinary S-equol, after which additional increases conferred limited benefit, indicating a potential saturation effect. However, this finding should be interpreted cautiously, as the present study was not designed or powered to formally test dose–response relationships. Further investigation in larger, suitably designed studies with sufficient numbers of S-equol producers will be required to confirm whether such a threshold truly exists and to establish the optimal dose for clinical benefit.

A multiple linear regression model adjusting for covariates such as age, dietary soy intake, skin phototype, and their interactions confirmed and extended these findings. At Day 42, S-equol remained a robust predictor of improved undereye wrinkle parameters and reduced TEWL. Interestingly, age emerged as a modulating factor, with older participants demonstrating more pronounced improvements in response to higher S-equol levels. This suggests potential synergism between chronological aging and the skin’s responsiveness to isoflavones, possibly due to age-related changes in estrogen receptor expression or skin barrier dynamics.

At Day 84, the predictive value of S-equol diminished, and age, soy intake, and skin phototype became more influential predictors. For instance, consuming Novasoy®400 by individuals with older ages and higher weekly soy intake were associated with improved wrinkle outcomes. These findings highlight the multifactorial nature of skin aging and the importance of considering individual characteristics and lifestyle factors when evaluating skin response to dietary interventions.

Interestingly, skin hydration showed a modest but statistically significant positive association with S-equol when data from both time points were combined, indicating a potential cumulative or delayed effect of isoflavone metabolites on stratum corneum water retention. This aligns with prior research suggesting that phytoestrogens can improve skin moisture by modulating epidermal barrier function and increasing hyaluronic acid synthesis (28, 29).

However, no consistent associations were found between S-equol levels and crow’s feet wrinkles across time points in our regression model, despite a 5.6% average reduction in crow’s feet wrinkle roughness (Ra) observed in the Novasoy®400 group at Day 84 compared to a 1.6% increase in the placebo group. This discrepancy may be due to the region-specific responsiveness of facial skin to phytoestrogens, with the under-eye area, where stronger S-equol associations were observed, being thinner and more vascularized than the crow’s feet region. Alternatively, the clinical improvement in crow’s feet may reflect cumulative effects not well captured by linear regression at fixed time points, especially in a small sample. It’s also plausible that wrinkle reduction depends on factors beyond circulating S-equol levels, such as localized estrogen receptor expression, skin biomechanical properties, or longer-term dermal remodeling that requires more than 12 weeks to manifest significantly. These findings emphasize the complexity of translating metabolite levels into visible skin outcomes and highlight the importance of selecting anatomically and biologically plausible endpoints in future trials.

Crow’s feet Ra was selected a priori as the primary endpoint because PRIMOS®3D measurements in this region offer high reproducibility and lower susceptibility to motion artefacts. Nonetheless, exploratory analyses indicated that under-eye wrinkle metrics—and TEWL—showed stronger associations with S-equol at Day 42, with attenuation by Day 84. This pattern may reflect a true biological plateau, but could also be influenced by behavioral variability, microbiome changes, or environmental factors such as UV exposure. Given the pilot design of the study, these findings should be regarded as hypothesis-generating. Future trials should therefore consider prioriti*z*ing under-eye parameters at earlier timepoints, include patient-reported outcomes to define clinical relevance, and stratify by S-equol producer status to enhance sensitivity, while also incorporating additional timepoints to better characteri*z*e the kinetics of any response. Previous studies have reported skin health benefits from soy isoflavone supplementation, but most lacked placebo controls, used multi-ingredient formulations, or focused on premenopausal or East Asian populations. Such designs limit the ability to isolate isoflavone effects. In contrast, our study tested soy isoflavones alone in a placebo-controlled setting, allowing clearer assessment of their impact. Unlike topical treatments that act on the upper skin layers, oral isoflavones may better target deeper dermal changes and higher-grade wrinkles, which we excluded, potentially explaining the limited results. Additionally, higher baseline soy intake and greater prevalence of S-equol producers in East Asian populations may underlie their more consistent benefits, further supporting the “equol hypothesis” that only S-equol producers experience measurable effects.

This study had several limitations that should be acknowledged:

First, as a pilot study, the trial may have been underpowered to detect meaningful effects, particularly in the exploratory analyses. Therefore, the results should be interpreted with caution. As previously noted, this study was designed as a pilot and, accordingly, no formal sample size calculation was performed. The achieved sample size was based on general recommendations for estimating variability in wrinkle measurements, but may be insufficient to provide adequate statistical power to detect small-to-moderate differences in the measured outcomes. As such, the findings should be viewed as exploratory and hypothesis-generating rather than confirmatory.

Second, we did not collect stool samples to evaluate the presence of S-equol producing bacterial species at baseline and therefore could not take into account S-equol producing status during randomisation. This may have resulted in S-equol producers being present in the placebo group who were inaccurately classified as non-producers, solely due to a lack of soy exposure during the study. This also prevented us from determining whether any women in the intervention group converted from non-producers to producers following Novasoy®400 intake. While we did evaluate habitual dietary soy intake at baseline, this cannot account for hidden soy consumption or early life exposure. A 2019 study of the French population reported that even soy non-eaters consumed 1.9 mg isoflavones per day, due to hidden soy in transformed foodstuffs (27). It is also well established that individuals who were regularly exposed to soy in early life are more likely to possess the gut bacteria to produce S-equol (17, 30). Future trials should establish S-equol status at baseline, incorporate it into stratification or subgroup analyses, and ideally collect stool samples to confirm the presence of equol-producing microbiota. In addition, it should be noted that baseline soy intake in our cohort was low (median 0.46 weekly servings), partially reflecting dietary patterns in Western populations, but limiting generalisability to other populations where soy consumption is substantially higher, e.g., east and south-east Asia. This context is important when interpreting our findings, as the skin benefits observed here may underestimate the magnitude of effect achievable in populations with higher habitual soy intake or greater prevalence of equol producers. Future studies should examine diverse populations, including those with higher baseline soy exposure, to better characterise the full range of isoflavone-related effects.

A third limitation to note is the inclusion of participants restricted to grades 2–5 wrinkles on Bazin’s scale. This choice was made to optimize measurement reproducibility, but in doing so it excluded women with more severe wrinkles who could feasibly derive greater benefit from soy isoflavone supplementation. It is possible that this design decision may have reduced our ability to observe an overall statistically significant treatment effect. Future studies should consider enrolling women across a wider range of wrinkle severities, to better understand whether greater efficacy is seen in those with more advanced skin ageing.

A fourth limitation to note relates to the clinical significance of the changes seen in this trial. The Novasoy®400 group showed a 5.6% reduction in crow’s feet wrinkle roughness compared with a 1.6% increase in the placebo group and this difference did not reach statistical significance. Moreover, the clinical significance of such a change is uncertain. There are currently no widely accepted thresholds for what constitutes a clinically meaningful improvement in PRIMOS-measured wrinkle parameters, and as such it is unclear whether changes of this magnitude would be perceptible or relevant to patients. Our findings should therefore be regarded as preliminary and hypothesis-generating, requiring confirmation in larger, longer studies that incorporate both objective measures and validated patient-reported outcomes.

## 5 Conclusion

Overall, this study underscores the potential of S-equol as a biomarker and mediator of skin health improvements following soy isoflavone supplementation, particularly in postmenopausal populations. Soy isoflavone supplementation increased urinary S-equol concentrations and was associated with improvements in under-eye wrinkle appearance, skin hydration, and barrier function—particularly among older postmenopausal women identified as S-equol producers. However, the modest variance explained by S-equol alone, and the attenuation of effects over time, highlight the need for multifactorial models incorporating age, soy intake, phototype, and other variables. These findings provide a compelling rationale for personalized nutritional strategies targeting skin aging, as well as further investigation into the temporal dynamics and mechanisms underlying phytoestrogen-related skin benefits.

## Data Availability

The original contributions presented in the study are included in the article/Supplementary material, further inquiries can be directed to the corresponding author/s.

## References

[1] Takuathung MN Na Takuathung M Teekachunhatean S Chansakaow S Klinjan P Inpan R. The effects of SOY extract nutraceuticals on postmenopausal women’s health: a randomized, double-blind, placebo-controlled trial. J Funct Foods. (2024) 113:106055. doi: 10.1016/j.jff.2024.106055

[2] Piérard GE Letawe C Dowlati A Piérard-Franchimont C. Effect of hormone replacement therapy for menopause on the mechanical properties of skin. J Am Geriatr Soc. (1995) 43:662–5. doi: 10.1111/j.1532-5415.1995.tb07202.x, PMID: 7775726

[3] Pivazyan L Avetisyan J Loshkareva M Abdurakhmanova A. Skin rejuvenation in women using menopausal hormone therapy: a systematic review and Meta-analysis. J Menopausal Med. (2023) 29:97. doi: 10.6118/jmm.22042, PMID: 38230593 PMC10796198

[4] Hamoda H Mukherjee A Morris E Baldeweg SE Jayasena CN Briggs P. ‘Joint position statement by the British menopause society, Royal College of Obstetricians and Gynaecologists and Society for Endocrinology on best practice recommendations for the care of women experiencing the menopause’, *post*. Reprod Health. (2022) 28:123–5. doi: 10.1177/20533691221104879 35603995

[5] Kim J-E Kang Y-G Seong Park J Lim T-G Won Lee K. Review of soybean phytochemicals and their bioactive properties relevant for skin health. J Food Nutr Res. (2017) 5:852–8. doi: 10.12691/jfnr-5-11-9

[6] Canivenc-Lavier M-C Bennetau-Pelissero C. Phytoestrogens and health effects. Nutrients. (2023) 15:317. doi: 10.3390/nu15020317, PMID: 36678189 PMC9864699

[7] Chen LR Ko NY Chen KH. Isoflavone supplements for menopausal women: a systematic review. Nutrients. (2019) 11:2649. doi: 10.3390/NU11112649 31689947 PMC6893524

[8] File SE Hartley DE Elsabagh S Duffy R Wiseman H. Cognitive improvement after 6 weeks of soy supplements in postmenopausal women is limited to frontal lobe function. Menopause. (2005) 12:18. doi: 10.1097/00042192-200512020-00014 15772567

[9] Li L Lv Y Xu L Zheng Q. Quantitative efficacy of soy isoflavones on menopausal hot flashes. Br J Clin Pharmacol. (2015) 79:593–604. Available at:. doi: 10.1111/bcp.12533, PMID: 25316502 PMC4386944

[10] Natarelli N Gahoonia N Maloh J Sivamani RK. Clinical efficacy of topical or oral soy supplementation in dermatology: a systematic review. J Clin Med. (2023) 12:4171. doi: 10.3390/jcm12124171, PMID: 37373864 PMC10299547

[11] Setchell KDR Brown NM Desai P Zimmer-Nechemias L Wolfe BE Brashear WT. Bioavailability of pure Isoflavones in healthy humans and analysis of commercial soy Isoflavone supplements. J Nutr. (2001) 131:1362S–75S. doi: 10.1093/jn/131.4.1362S, PMID: 11285356

[12] Muthyala RS Ju YH Sheng S Williams LD Doerge DR Katzenellenbogen BS. Equol, a natural estrogenic metabolite from soy isoflavones: convenient preparation and resolution of R- and S-equols and their differing binding and biological activity through estrogen receptors alpha and beta. Bioorg Med Chem. (2004) 12:1559–67. doi: 10.1016/j.bmc.2003.11.035, PMID: 15018930

[13] Sekikawa A Wharton W Butts B Veliky CV Garfein J Li J. Potential protective mechanisms of S-equol, a metabolite of soy Isoflavone by the gut microbiome, on cognitive decline and dementia. Int J Mol Sci. (2022) 23:11921. doi: 10.3390/ijms231911921, PMID: 36233223 PMC9570153

[14] Lv J Jin S Zhang Y Zhou Y Li M Feng N. Equol: a metabolite of gut microbiota with potential antitumor effects. Gut Pathogens. (2024) 16:35. doi: 10.1186/s13099-024-00625-9, PMID: 38972976 PMC11229234

[15] Schröder C Matthies A Engst W Blaut M Braune A. Identification and expression of genes involved in the conversion of daidzein and genistein by the equol-forming bacterium *slackia isoflavoniconvertens*. Appl Environ Microbiol. (2013) 79:3494–502. doi: 10.1128/AEM.03693-12, PMID: 23542626 PMC3648055

[16] Yokoyama SI Suzuki T. Isolation and characterization of a novel equol-producing bacterium from human feces. Biosci Biotechnol Biochem. (2008) 72:329. doi: 10.1271/bbb.80329 18838805

[17] Jackson RL Greiwe JS Schwen RJ. Emerging evidence of the health benefits of S-equol, an estrogen receptor β agonist. Nutr Rev. (2011) 69:432–48. doi: 10.1111/j.1753-4887.2011.00400.x, PMID: 21790611

[18] Setchell KDR Brown NM Lydeking-Olsen E. The clinical importance of the metabolite Equol—a clue to the effectiveness of soy and its Isoflavones. J Nutr. (2002) 132:3577–84. doi: 10.1093/jn/132.12.3577, PMID: 12468591

[19] Oyama A Ueno T Uchiyama S Aihara T Miyake A Kondo S. The effects of natural S-equol supplementation on skin aging in postmenopausal women: a pilot randomized placebo-controlled trial. Menopause. (2012) 19:202–10. doi: 10.1097/gme.0b013e318227427b 21934634

[20] Wu J Oka J Ezaki J Ohtomo T Ueno T Uchiyama S. Possible role of equol status in the effects of isoflavone on bone and fat mass in postmenopausal Japanese women: a double-blind, randomized, controlled trial. Menopause. (2007) 14:866–74. doi: 10.1097/gme.0b013e3180305299 17464237

[21] Frankenfeld CL Patterson RE Horner NK Neuhouser ML Skor HE Kalhorn TF. Validation of a soy food-frequency questionnaire and evaluation of correlates of plasma isoflavone concentrations in postmenopausal women. Am J Clin Nutr. (2003) 77:674–80. doi: 10.1093/ajcn/77.3.674, PMID: 12600860

[22] Saha S Kroon PA. A simple and rapid LC-MS/MS method for quantification of Total Daidzein, Genistein, and Equol in human urine. J. Anal. Methods Chem. (2020) 2020:1–9. doi: 10.1155/2020/2359397, PMID: 32399306 PMC7201686

[23] Palma-Duran SA Caire-Juvera G Campa-Siqueiros MM Chávez-Suárez KM Robles-Burgueño MdR Gutiérrez-Coronado ML. A comprehensive HPLC-DAD-ESI-MS validated method for the quantification of 16 phytoestrogens in food, serum and urine. Appl Sci. (2020) 10:8147. doi: 10.3390/app10228147

[24] Sim J Lewis M. The size of a pilot study for a clinical trial should be calculated in relation to considerations of precision and efficiency. J Clin Epidemiol. (2012) 65:301–8. doi: 10.1016/j.jclinepi.2011.07.011, PMID: 22169081

[25] On Harmonisation E9 Expert Working Group, I.C. ICH harmonised tripartite guideline. Statistical principles for clinical trials. Stat Med. (1999) 18:1905–42. 10532877

[26] R Core Team. A language and environment for statistical computing. R Foundation for Statistical Computing, Vienna, Austria. (2021). Available online at: https://www.R-project.org/

[27] Lee A Beaubernard L Lamothe V Bennetau-Pelissero C. New evaluation of Isoflavone exposure in the French population. Nutrients. (2019) 11:2308. doi: 10.3390/nu11102308, PMID: 31569435 PMC6835759

[28] Rizzo J Min M Adnan S Afzal N Maloh J Chambers CJ. Soy protein containing isoflavones improves facial signs of photoaging and skin hydration in postmenopausal women: results of a prospective randomized double-blind controlled trial. Nutrients. (2023) 15:4113. doi: 10.3390/nu15194113, PMID: 37836398 PMC10574417

[29] Patriarca MT Barbosa de Moraes AR Nader HB Petri V Maciel Martins JR Teixeira Gomes RC. Hyaluronic acid concentration in postmenopausal facial skin after topical estradiol and genistein treatment: a double-blind, randomized clinical trial of efficacy. Menopause. (2013) 20:898. doi: 10.1097/GME.0b013e318269898c 23435032

[30] Crawford SL Jackson EA Churchill L Lampe JW Leung K Ockene JK. Impact of dose, frequency of administration, and equol production on efficacy of isoflavones for menopausal hot flashes: a pilot randomized trial. Menopause. (2013) 20:1–21. doi: 10.1097/GME.0b013e3182829413 23511704 PMC3723773

