# Supplementary material for: S-equol status modulates skin response to soy isoflavones in postmenopausal women: results from a randomized placebo-controlled pilot trial
Source: Front Nutr. 2025 Nov 3;12:1671835. doi: 10.3389/fnut.2025.1671835 (PMC12621142; doi:10.3389/fnut.2025.1671835)
Supplement: Supplementary file 1 [file Table_1.docx]

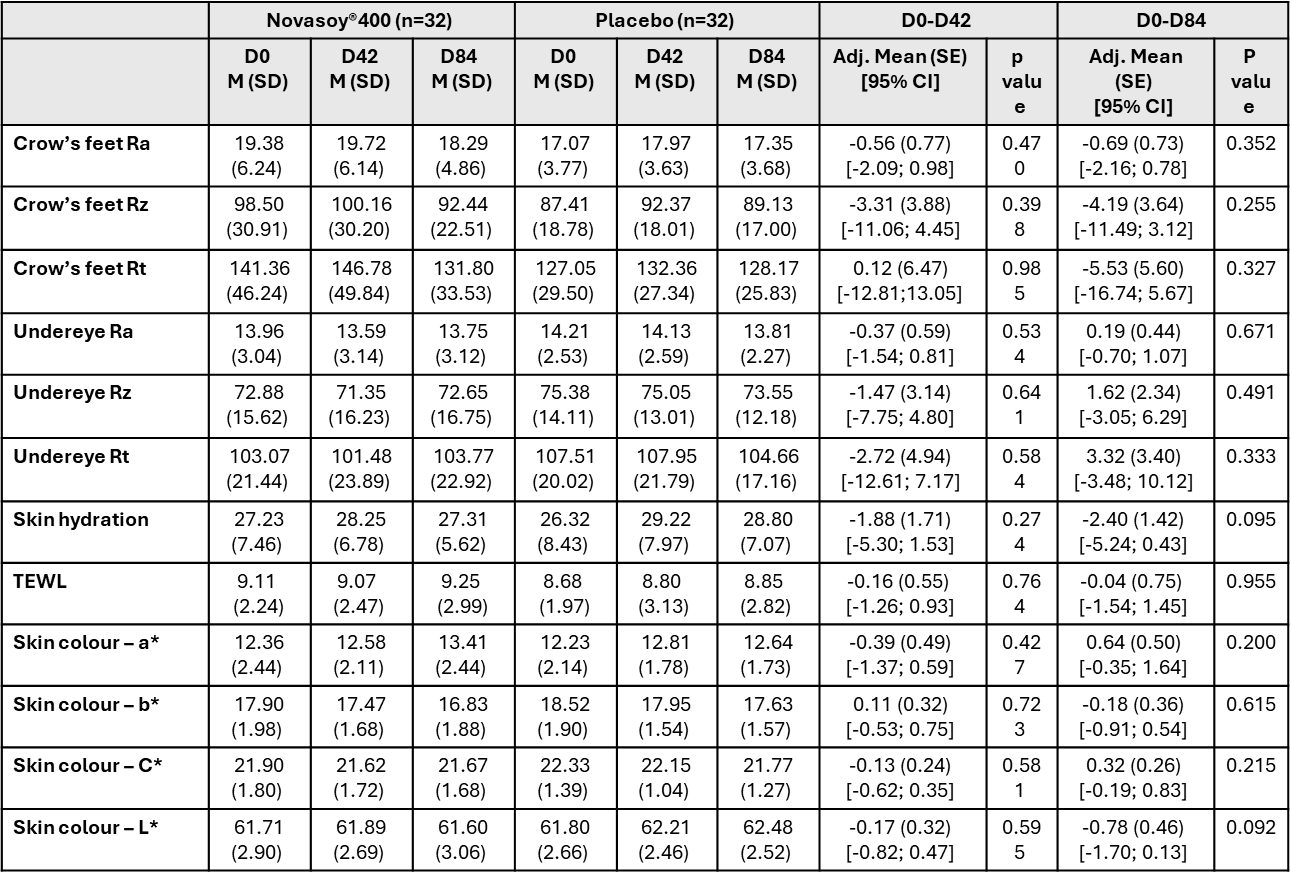
**Supplementary** **Table 1. Measures of skin parameters by timepoint and intervention group in the ITT dataset***Ra - average roughness, Rz - average height of the roughness/ average relief, Rt - maximum height of the roughness/ maximum relief amplitude, TEWL- trans epidermal water loss, L*- clarity (from dark to light), a* - green-to-red spectrum, b*: blue-to-yellow spectrum, C* - saturation; Adjusted  Means, Standard Error, 95%CI and p-values from mixed linear models of the ITT dataset.*

|  |  | **D0** | **D42** | **D84** | **p-value (D42vsD0)** | **p-value (D84vsD0)** |
| --- | --- | --- | --- | --- | --- | --- |
| **Genistein** | NovaSoy | 0.15±0.06 | 4.71±6.35* | 4.55±7.03* | <0.001 | <0.001 |
|  | Placebo | 0.38±0.58 | 1.26±2.27 | 0.57±0.93 | 0.905 | 1 |
| **Daidzein** | NovaSoy | 0.34±0.39 | 8.37±15.33* | 6.99±8.85* | <0.001 | <0.001 |
|  | Placebo | 0.44±0.69 | 0.96±1.69 | 0.42±0.77 | 0.205 | 0.543 |
| **Dihydrodaidzein** | NovaSoy | 0.07±0.05 | 2.57±3.57 | 1.68±1.6* | 0.043 | 0.023 |
|  | Placebo | 0.29±0.44 | 0.97±1.71 | 0.39±0.43 | 0.606 | 0.463 |
| **S‐equol** | NovaSoy | 0.42±0.00 | 8.1±6.55 | 9±10.64* | NA^#^ | NA^#^ |
|  | Placebo | 2.2±0.37 | 7.81±5.7 | 5.76±11.26 | 0.857 | 0.381 |

**Supplementary Table 2.** Average concentration (ppm) in subjects that have any detectable amount of urine metabolites (genistein, daidzein, dihydrodaidzein and S‐equol) and their standard deviation, at Day 0, Day 42 and Day 84. Statistical significance between different time points and the baseline (D0) was assessed using the Mann-Whitney-Wilcoxon test. Also, statistical significance between Novasoy®400 and placebo groups at each time point was indicated with asterisks when significant. (*) p-value <0.05. (^#^) Statistical analysis not applicable as at baseline we had only one participant producing S-equol in the Novasoy®400 group.


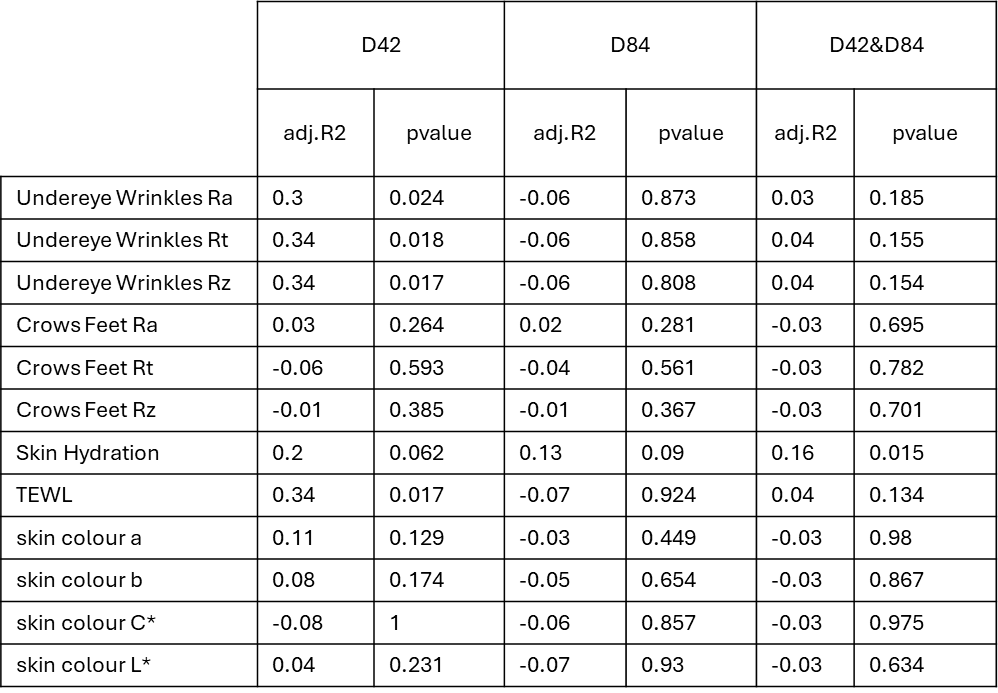


**Supplementary Table 3**: **Results of the simple regression analysis of each skin parameter against concentration of S-equol in urine at days 42, 84 and overtime (D42&D84).** Data includes women that were used in the multiple regression model and had any detectable amount of S-equol in that time point (n_D42_=14, n_D84_=17, n_D42&D84_=31). Table includes adjusted R² and p-values of the model obtained with an F-test.


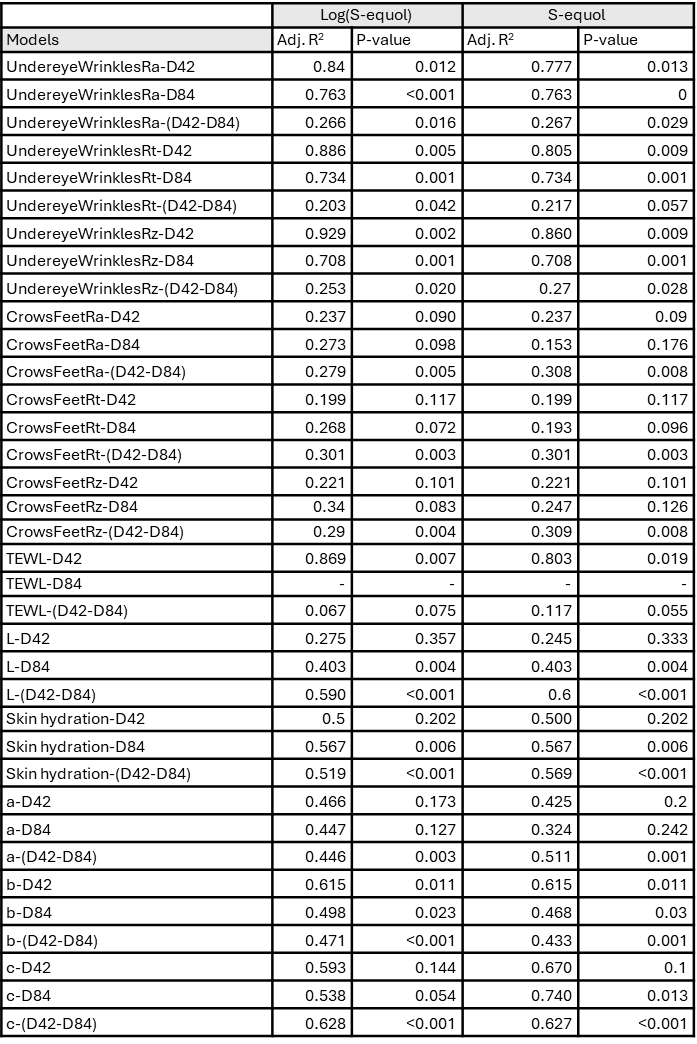


**Supplementary Table 4.**Comparison of the results obtained with the multiple regression model using a non-transformed concentration of S-equol in urine or the log-transformed S-equol, for all skin parameters at days 42, 84 and overtime (D42&D84). Adjusted R² and p-values were used to determine the best S-equol transformation in each case. In the case of TEWL at day 84, none of the independent variables in the model significantly contribute to explain its variance.


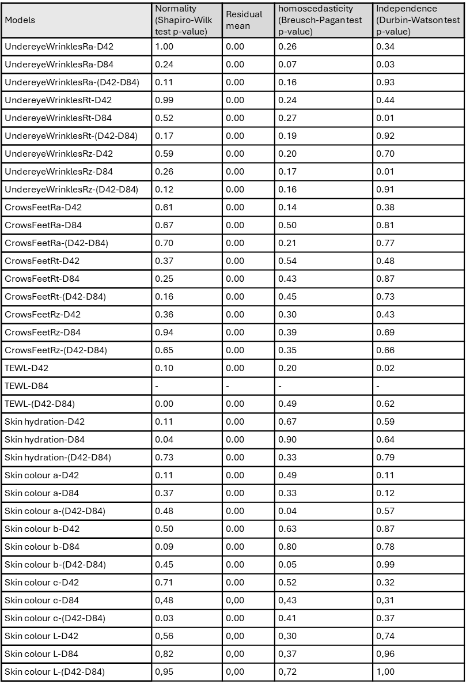


**Supplementary Table 5.** This table summarizes the results of statistical tests used to evaluate key assumptions of linear regression models. Normality of residuals was assessed using the Shapiro-Wilk test; p < 0.05 indicates deviation from normality. Residual mean values are reported to assess model bias; values close to zero suggest unbiased residuals. Homoscedasticity (constant variance) was tested using the Breusch-Pagan test; p < 0.05 suggests heteroskedasticity. Independence of residuals was evaluated using the Durbin-Watson statistic, where p < 0.05 suggests may indicate positive autocorrelation.
